# Ubiquitin-Like Protein from Human Placental Extract Exhibits Collagenase Activity

**DOI:** 10.1371/journal.pone.0059585

**Published:** 2013-03-26

**Authors:** Debashree De, Piyali Datta Chakraborty, Jyotirmoy Mitra, Kanika Sharma, Somnath Mandal, Aneesha Das, Saikat Chakrabarti, Debasish Bhattacharyya

**Affiliations:** 1 Division of Structural Biology and Bioinformatics, Council of Scientific and Industrial Research - Indian Institute of Chemical Biology, Calcutta, West Bengal, India; 2 Research and Development, Albert David Ltd., Calcutta, West Bengal, India; Chang Gung University, Taiwan

## Abstract

An aqueous extract of human placenta exhibits strong gelatinase/collagenase activity in zymography. 2-D gel electrophoresis of the extract with gelatin zymography in the second dimension displayed a single spot, identified as ubiquitin-like component upon MALDI/TOF MS/MS analysis. Immunoblot indicated presence of ubiquitin and absence of collagenase in the extract. Collagenase activity of the ubiquitin-like component was confirmed from the change in solubility of collagen in aqueous buffer, degradation of collagen by size-exclusion HPLC and atomic force microscopy. Quantification with DQ-gelatin showed that the extract contains 0.04 U/ml of collagenase activity that was inhibited up to 95% by ubiquitin antibody. Ubiquitin from bovine erythrocytes demonstrated mild collagenase activity. Bioinformatics studies suggest that placental ubiquitin and collagenase follow structurally divergent evolution. This thermostable intrinsic collagenase activity of placental extract might have wide physiological relevance in degrading and remodeling collagen as it is used as a drug for wound healing and pelvic inflammatory diseases.

## Introduction

Investigations over the years have enriched our understanding of the dynamics of acute wound healing which is brought about by the interplay of a large number of regulatory molecules and cellular mediators. Acute wound healing follows an orderly cascade of events, which can broadly be categorized into three interrelated dynamic phases; namely inflammatory or exudative, proliferative, regenerative and remodeling [1]-[4]. A number of extracellular and cell surface proteases, in coordination with various regulatory molecules such as cytokines and growth factors, coordinates the degradation and synthesis of the extracellular matrix to ensure effective healing [5]-[7]. Of the various proteases, zinc-dependent matrix metalloproteases (MMPs) from the metzincin superfamily direct wound healing by controlling platelet aggregation, macrophage and neutrophil function, cell migration and proliferation, angiogenesis and collagen secretion, and extracellular matrix remodeling [5], [8]-[10]. The MMP family can be divided into subgroups of collagenases, gelatinases, stromelysins and membrane-type MMPs (MT-MMPs) based on their substrate specificity and structure. Members of the collagenase subfamily – collagenase-1 (MMP-1), collagenase-2 (MMP-8), and collagenase-3 (MMP-13) – have the unique ability to cleave native fibrillar collagens of type I, II, and III at a specific site generating N- and C-terminal fragments, of lengths approximately in the ratio 3∶1, which denature at 37°C and are further degraded by other MMPs, such as gelatinases and non-specific proteases [11], [12].

Proteases and their inhibitors regulate the balance between tissue degradation and regeneration. Slow removal of necrotic tissue delays the onset of healing while excess of proteolytic activity destroys growth factors and their receptors, as well as inhibit angiogenesis and breakdown granulation tissue resulting in tissue damage [8], [13], [14]. Chronic wound is characterized by the presence of necrotic tissue and efficient debridement is necessary to accelerate healing [2]. Enzymatic debridement ensures effective healing without the need for surgical intervention [15]. An aqueous extract of human placenta has shown to be effective in chronic non-healing wounds by virtue of its debriding, anti-inflammatory and anti-microbial properties [16]. The manufacture of the extract is well standardized [17]. Biochemical characterization of the extract has led to the identification of fibronectin type-III like peptide and functional NADPH [18], [19]. In addition, the extract exhibits anti-microbial property against commonly occurring pathological micro-organisms [20], ability for *in vitro* NO induction by mouse peritoneal macrophages [21] and enhancement of cell adhesion [22].

Recently a peptide fraction isolated from the placental extract showed ability to stabilize trypsin activity possibly through fibronectin type III-like peptide [23]. Considering the importance of proteases in wound healing and concomitant to previous findings of the stability of trypsin, attempts were made to verify if the peptide fraction exhibited similar effect on collagenases or MMPs. Results indicated that the extract itself displayed distinct proteolytic activity, a phenomenon unexpected as the extract is manufactured under elevated conditions of temperature and pressure where most of the enzymes are irreversibly inactivated. The component responsible for the proteolytic activity was identified to be an ubiquitin-like protein. This study, therefore, reports for the first time that an ubiquitin-like component from human placental extract exhibits distinct collagenase activity. In addition, a pool of ubiquitin-like peptides with no proteolytic activity has also been identified in the extract. It is already known that ubiquitin and ubiquitin-like proteins (Ubls), collectively known as ‘ubiquitons’, perform a host of important functions such as protein degradation, antigen processing, apoptosis, biogenesis of organelles, cell cycle and division, fertilization and gametogenesis, DNA transcription and repair, differentiation and development, signaling, immune response and repair [24]-[31]. Though verification of these properties in the placental extract is not within the purview of the current study, the relevance of this finding lies in the fact that these ubiquitin-like peptides in the extract might have important implications in wound healing and explains one of the mechanisms by which the extract possibly functions.

## Results

### Human Placental Peptides Show Gelatinase/Collagenase Activity

**Figure 1:**
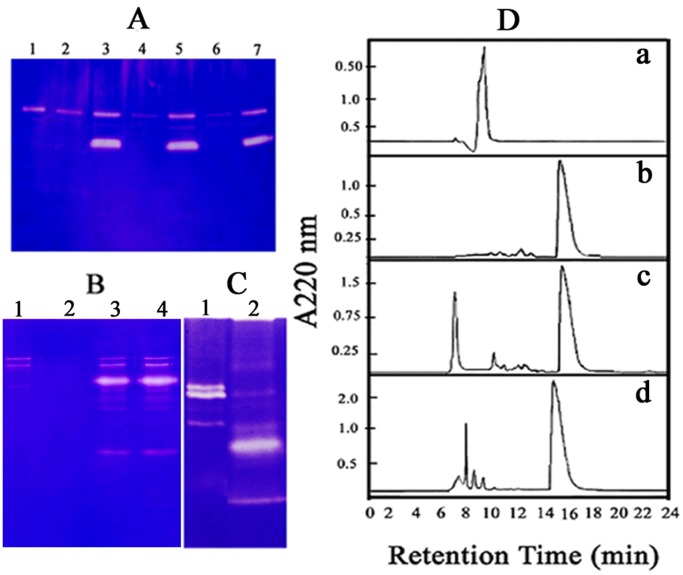
Gelatinase/Collagenase activity of Placental Extract. (A) Gelatin Zymography - Lane 1, MMP 2; lanes 2, 4 and 6, MMP 2 activation profile in presence of APMA for 0, 1 and 2 hrs; lanes 3, 5 and7, MMP 2 incubated with peptide fraction at 37°C for same duration. (B) Gelatin Zymography - Lane 1 and 2, 0.1 ng collagenase incubated at pH 7.5 for 0 and 20 h at 37°C; lanes 3 and 4, 20 µg peptide fraction with 0.1 ng bacterial collagenase incubated for 0 and 20 h. (C) Collagen zymography. Lanes 1 and 2, 1.0 and 0.1 ng of collagenase; Lane 3, 20 µg of placental peptide fraction. (D) SE-HPLC analysis of collagenase activity of placental extract. a: collagen (R_t_ = 9.66±0.05 min, n = 5), b: placental extract (15.46±0.08 min), c : collagen treated with the extract at 0 h (major components at 7.3±0.03 and 16.1±0.08 min) and d : 168 h (major components at 7.56±0.01, 8.20±0.03, 8.83±0.001 and 9.60±0.04 min) respectively (n = 5).

This study originated to verify regulation (stabilization or activation/inhibition) of MMP or bacterial collagenase by the peptides isolated from human placenta. Although no regulatory effect was observed, additional protease bands other than the MMPs and collagenase were detected in the gelatin zymography (Figure 1A and B). These additional bands appeared irrespective of duration of incubation and thus were not generated from the MMPs. Further, these bands also appeared in absence of the MMPs (Figure 1A) and its intensity was proportional to the amount of the placental peptide applied (50–500×diluted). These findings indicated that the isolated peptide fraction itself exhibited protease activity.

The source of the test material being placenta, there remains a possibility of blood protease content in it. A zymogram was developed with the placental peptides along with blood serum and plasma. The placental protease displayed bands distinctly different from the blood proteases [32]. Further, serine and metalloproteases being dominant in blood, the peptide fraction (10×diluted) was treated with 0.5 mM phenylmethyl sulphonyl fluoride (PMSF; a serine proteases inhibitor) or 4 mM Ca^2+^ (an activator of metalloproteases) separately for 10 min at 25°C [33]. Results indicated no change in the band intensities of the peptide fraction incubated with either PMSF or Ca^2+^.

Protease assay of the peptide fraction using azoalbumin or azocaesin as substrates did not demonstrate detectable activity. In BSA zymography, the peptide fraction indicated no band. A relatively sensitive technique for identification of trichloroacetic acid (TCA) insoluble larger fragments generated after hydrolysis of large protein substrates is SDS-PAGE. BSA or casein incubated at 37°C for 48 h in presence of the peptide fraction did not present any significant fragmentation in SDS-PAGE.

Considering the narrow specificity displayed by placental protease and gelatin being denatured collagen, collagen zymography was performed. Results indicated presence of one major and a few minor collagenase bands in the peptide fraction but all were distinctly different from bacterial collagenase (Figure 1C). Thus, collagenase activity by the isolated peptide fraction was indicative. Appearance of a low intensity multiple banding patterns indicated self-association/aggregation of single functional peptide/s or involvement of multiple peptides. Fibronectin type III-like peptide is an important constituent of placental extract. Gelatin zymography of human fibronectin type III-like peptide, however, did not exhibit any detectable band.

Collagenase activity of placental extract was also followed using Size-Exclusion High Performance Liquid Chromatography (SE-HPLC). Collagen and the placental extract showed R_t_ (retention time) of 9.66±0.05 and 15.46±0.08 min respectively (n = 5 each) (Figure 1D, a-d). A solution of collagen and the placental extract when applied at 0 h of mixing demonstrated two peaks of R_t_ 7.3±0.03 and 16.1±0.08 min respectively (n = 5). The shift in corresponding R_t_ was possibly due to binding of components of the extract with collagen (Figure 1D, c). After 72 h of incubation, the collagen peak was reduced to four components with R_t_ 7.56±0.01, 8.20±0.03, 8.83±0.001 and 9.60±0.04 min. The placental peptide peak was, however, retained (Figure 1D, d). Collagen incubated with ubiquitin (bovine erythrocytes) did not present significant degradation.

### Identification of Gelatinase/Collagenase Component

**Figure 2:**
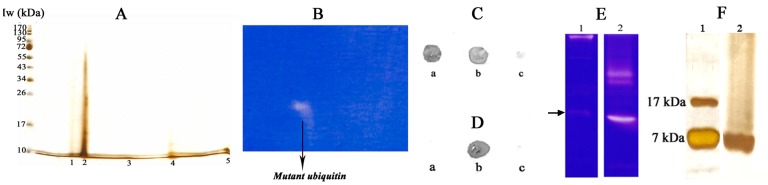
Identification of the component responsible for collagenase activity. (A) 2-D gel electrophoresis of placental extract (100x). Positions of Mw markers are indicated. (B) Gelatin zymography in the second dimension. The activity corresponded to spot number 3 of A. (C) Immunological cross-reactivity between anti-sera of ubiquitin and (a) ubiquitin, (b) peptide fraction and (c) ovalbumin. (D) Immunological cross-reactivity between anti-sera of peptide fraction and (a) collagenase, (b) peptide fraction and (c) ovalbumin. (E) Gelatin zymography. Lane 1, ubiquitin (bovine); and Lane 2, peptide fraction. The arrow indicates the faint band of ubiquitin which roughly corresponds to that of placental ubiquitin. (F) Silver stained SDS-PAGE of ubiquitin (bovine, lane 1) and peptide fraction (lane 2). Approximate Mw of ubiquitin bands have been indicated.

Profiling of the peptide fraction by 2-D gel electrophoresis was carried out to ascertain the number of components present in the peptide fraction and to identify them. Electrophoresis followed by silver staining indicated presence of five components of pIs 4.29, 4.67, 6.34, 7.82 and 9.84 of ∼ 7 kDa (spot numbers 1–5, Figure 2A). The spots analyzed by MALDI ToF/ToF combined with Mascot Search indicated presence of four variants of mutant human ubiquitin and hemoglobin (Table 1). Three components with the same accession code observed in the 2-D gel was possibly owing to mutations of ubiquitin in regions which have not been reflected in the derived sequences in Mascot Search; or short truncation at N- or C- terminal ends affecting its pI but not mass and hence not sensitive enough to be detected by SDS-PAGE; or modification of ubiquitin during the manufacture of the extract. Identification of the protease was done by 2-D gel electrophoresis with gelatin zymography in the second dimension. Results indicated that of the 5 components identified in Figure 2A, only one showed gelatinase activity (Figure 2B). This peptide corresponded to spot number 2 (Figure 2A).

**Table 1:** Assignment of the peptides isolated from the spots of the 2-D gel electrophoresis (Figure 2 A) by MS/MS and MASCOT.

Spot No.	Peptide	Accession Code	Homology with A Protein from	Mascot Score	Matched Peptide Fragments	MS/MS Derived Sequence
2.	Hemoglobin mutant CHAIN B,D, C93A,C112G deoxy, chain B	1GBUB	Human	62	3	R. LLVVYPWTQR.FK. GTFATLSELHADKK. LHVDPENFR. L
3.	1d8 ubiquitin mutant YES	1C3TA	Human	74	3	K TLTVELEPSDTVEK. EGIPPDQQR. LK. ESTIHLVLR. L
4.	Ubiquitin C Splice variant	Q5UGI3	Human	134	6	K. EGIPPDQQR. LR. TLSDYNIQK. EK. ESTLHLVLR. LK. TITLEVEPSDTIEK. EGIPPDQQR. LK. ESTLHLVLR. L
5.	Ubiquitin C Splice variant	Q5UGI3	Human	131	6	K. EGIPPDQQR. LR. TLSDYNIQK. EK. ESTLHLVLR. LK. TITLEVEPSDTIEK. EGIPPDQQR. LK. ESTLHLVLR. L
6.	Ubiquitin C Splice variant	Q5UGI3	Human	136	6	K. EGIPPDQQR. LR. TLSDYNIQK. EK. ESTLHLVLR. LK. TITLEVEPSDTIEK. EGIPPDQQR. LK. ESTLHLVLR. L

### Immunoblot

The presence of ubiquitin in the peptide fraction was confirmed by immunoblot using ubiquitin antibody against peptide fraction, bovine ubiquitin and lysozyme. Placental peptide and bovine ubiquitin demonstrated distinct spots, validating the mass spectrometric data (Figure 2C). Lysozyme being a negative control, exhibited no spot. To exclude the possibility of contamination of collagenase or its functional fragment in the placental peptide pool, immunoblot was performed with the anti-sera of placental peptide against collagenase, placental peptide and ovalbumin (negative control). Results indicated that only the peptide fraction strongly cross-reacted whereas neither collagenase nor ovalbumin responded (Figure 2D).

### Comparison between Bovine and Placental Ubiquitin

Bovine ubiquitin was compared with the peptide fraction by gelatin zymography and SDS-PAGE. Gelatin zymography with 20 µg bovine ubiquitin displayed a distinct band at the top along with a few relatively faint bands. The peptide fraction showed a distinct band along with other diffused bands, possibly owing to aggregation. Presence of a proteolytic band in bovine ubiquitin roughly corresponding to placental protease has been indicated (Figure 2E). A 10% SDS-PAGE followed by silver staining of bovine ubiquitin reflected bands of Mw 7 and 17 kDa, the later being covalently linked dimeric form of the monomer. The placental peptide pool exhibited unresolved mass of 7 kDa (Figure 2F).

### Gelatinase/Collagenase Assay

Though the placental peptide fraction demonstrates strong band in gelatin zymography, it did not apparently respond to DQ-gelatinase/collagenase assay maintaining the recommended protocol. Incubation of the peptide fraction in buffer for 72 h at 37°C restored the activity maximally (Figure S1A tracing a). Placental extract exhibited 0.04 U/ml of collagenase activity. Treatment with 200× dilute ubiquitin antibody inhibited the activity by 95.0%, indicating that the gelatinase/collagenase activity is solely exhibited by placental ubiquitin (Figure S1A tracing b). The time course of activation of the placental ubiquitin indicated that between 24–48 h the activation was complete, which remained stable up to at least 72 h (Figure S1B).

### Physical Evidences of Collagenase Activity

By definition, collagenases are endopeptidases that digest collagen under native conformational state in the triple helix region [34]. Collagenase activity of the peptide fraction was further reflected when collagen (2 mg/ml) was incubated in presence of the extract in 25 mM Na-phosphate, pH 7.5 at 37°C. Results were compared with appropriate controls where collagen was incubated under identical conditions in presence of collagenase and bovine ubiquitin. Collagen in buffer was not soluble and hence remained as a clump. Collagen in presence of the extract displayed increased solubility with time and completely disappeared after 168 h, while the control sets containing only collagen, or collagen incubated with bovine ubiquitin and collagen incubated with collagenase remained intact (Figure S2).

Atomic Force Microscopy (AFM) was used to visualize the integrity of collagen in presence of placental peptides. Adsorbed collagen assemblies were imaged by AAC mode in fluid using a 100 µm scanner. Control collagen prepared at 4°C displayed mature fibrillar networks, with an average diameter of 2 µm and length greater than 50 µm. These mature collagen fibrils assemble to form the network. The mature fibrils appeared larger centrally with tapered ends displaying declining slopes. Collagen monomers usually assemble to form cable-like structures with varying diameters between 10–500 nm. An enlarged view of the network showed individual monomer filaments with an average diameter of approximately 431.2 nm, complying with standard findings [35].

**Figure 3:**
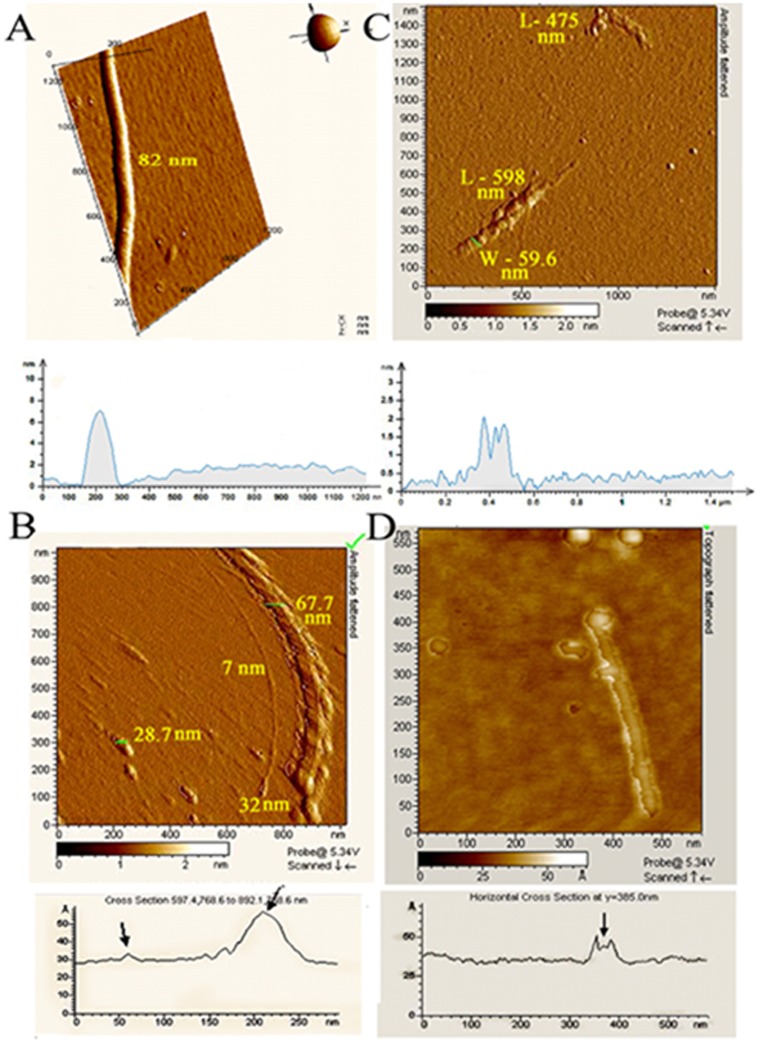
AFM of collagen. (A) Enlarged 3D view of collagen monomer filament (diameter 82±10 nm). (Lower Panel) Cross-section indicating intact collagen filament. (B) Amplitude flattened image of collagen (67.7 nm) incubated with collagenase. A fiber (7 nm) appearing from its side is also apparent. The bulge diameter of 32 nm is similar to another bulge (∼28.7 nm). (Lower Panel) Loss in integrity of collagen. The arrow on the left indicates the 7 nm fiber while that on the right indicates the 67.7 nm fiber. (C) Amplitude flattened image of collagen incubated with peptide fraction indicated two fragments of lengths 598 and 475 nm and width 59.6 nm each. (Lower Panel) Loss in integrity and fragmentation of collagen. (D) Topograph flattened image of collagen treated with ubiquitin indicates a single particle with a bulging head; scattered bulges are also apparent. (Lower Panel) Loss in integrity of collagen. The arrow indicates poor peak shape of the collagen and the possible region of wearing out from this molecule. In all the figures presented, a color scale bar indicating sample height from the mica sheet has been represented. The dark shade indicates depression of the sample while the white color represents maximum elevation.

Control collagen incubated at 37°C for 48 h when imaged in liquid mode exhibited intact monomeric filament of 82 nm, as evident from the cross-section (Figure 3A) [35]. Image of collagen treated with collagenase indicated gradual loss in integrity of the fibril. The monomer filament measured approximately 67.7 nm in diameter and beside it, a very thin fiber-like structure was apparent with a diameter of 7 nm. Careful observation indicated that this fiber possibly generated from the monomeric filament, with another fiber emerging from its side. Additionally, the molecule appeared to bulge from the bottom. The rest of the area appeared to be covered by these bulges protruding from the tapering end of the monomer filament (Figure 3B). Collagen treated with the peptide fraction indicated fragments of different lengths and diameters accompanied by loss in integrity of the fibril. The rest of the area showed small globular particles possibly originating from the denatured peptide fragments. An enlarged collagen monomer filament indicated fragmentation followed by denaturation. Loss in integrity is evident from the cross-section of the filament (Figure 3C). Finally, image of collagen treated with bovine ubiquitin demonstrated fragments of different lengths and diameters. Upon further magnification, a collagen molecule with a bulging head was apparent. The cross-section clearly indicated loss in integrity of the molecule. The bulges appear to protrude from the molecule, which further disintegrate (Figure 3D).

## Discussion

The present study reports that an ubiquitin-like protein present in human placental extract exhibits intrinsic collagenase activity. This finding has been supported by collagen zymography, 2-D gel electrophoresis followed by MALDI/ToF analysis, DQ-gelatinase/collagenase assay and physical methods such as change in solubility of collagen and AFM. In collagen zymography, exchange of SDS by Triton X-100 restores in part the original triple-helical conformation of collagen, allowing its degradation specifically by collagenases [36], [37]. Absent or inadequate bands observed in BSA zymography and SDS-PAGE indicated that placental peptide fraction has partial or no effect on common protease substrates and is of narrow specificity [38]. The collagenase activity was identified to be a human ubiquitin-like component by 2-D gel electrophoresis of the peptide fraction coupled with MALDI analysis and Mascot search (Table 1). Immunoblot experiments in addition indicated that an ubiquitin-like and no collagenase-like component was present in the extract (Figure 2C, D). Collagenase assay using DQ-gelatin as substrate (Figure S1A, B) indicated that the extract exhibited 0.04 U/ml of collagenase activity which demonstrated an initial lag phase of 24 h, following which, it got maximally activated between 24–48 h and remained stable at least for 72 h (Figure S1B). This observation suggested that possibly some stabilizer binds to and inactivates the enzyme, which upon incubation at 37°C for 24 h is activated. However, incubation of the peptide fraction between 50–90°C followed by immediate assay or incubation at 25°C to ensure reversible thermal unfolding of the component or alternately using 2–6 M guanidium hydrochloride could not induce activation to reduce the lag phase. A time dependent conformation change or dissociation of a ligand from the ubiquitin-like component cannot be ruled out. Nonetheless, the involvement of ubiquitin-like component was supported by up to 95% inactivation in presence of ubiquitin antibody (Figure S1A).

As already mentioned, a protease qualifies to be a collagenase when it has the ability to cleave native triple helical collagen [34], [39]. Collagen incubated in presence of the extract was solubilized indicating proteolysis (Figure S2). Collagenase being large, could not cleave native, insoluble collagen efficiently as compared to placental ubiquitin of small size. Bovine ubiquitin did not present detectable fragmentation possibly because its activity is not as strong as compared to the placental ubiquitin. Gelatin zymography being more sensitive, bovine ubiquitin indicated a distinct band different from that of the placental component (Figure 2E). Further, direct evidence of collagenase activity of the placental ubiquitin was made by AFM wherein loss in collagen network, general fragmentation and bulge-like appearance owing to denaturation and aggregation compared to controls were observed (Figure 3).

Ubiquitin mediated protein degradation is possibly one of the most well studied mechanisms of post-translational protein regulation in eukaryotes [24]. Increasing evidence suggests that ubiquitons also play roles distinct to protein degradation [25]-[31]. In addition, ubiquitin purified from either human or buffalo erythrocytes show intrinsic proteolytic activity against β-galactosidase or myoglobin with different kinetic behavior [38], [40]. The identified activity was heat-labile and exhibited autodigestion. Though it was comparable to other proteolytic enzymes, the ubiquitins could not be assigned under any family or group with certainty. The present study reports that placental ubiquitin exhibits intrinsic collagenase activity, distinct from that previously reported not only in terms of its kinetics but also stability and specificity. The novelty of this finding is that despite the manufacture of the extract at high temperature and pressure, the ubiquitin-like component retains the collagenase activity. Three possibilities in support of this finding have been proposed. First, ubiquitin is a very stable molecule with a rich secondary and tertiary structure and has a pronounced hydrophobic core [41]. Second, the identified ubiquitin in the placental extract being a variant of the standard ubiquitin is resistant to thermal denaturation. Third, in many instances peptides as aggregated mass confer stability [42].

**Figure 4:**
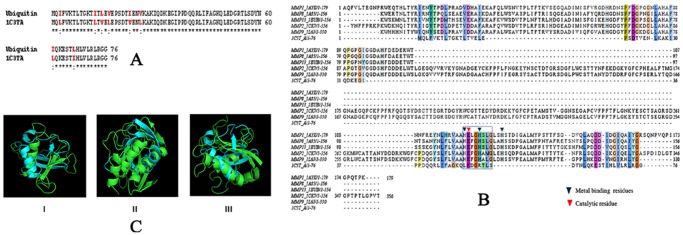
Comparison between ubiquitin and collagenase. (A) 2.0.12 ClustalW Sequence alignment between human ubiquitin and 1C3TA (mutant ubiquitin). The ‘*’ and ‘:’indicates identical and highly similar residues respectively. The mutated sequences have been highlighted in bold-red, which are also highly similar to corresponding ubiquitin amino acid residues. (B) Multiple sequence alignment of the peptidase domains of the matrix metalloproteases, obtained from their corresponding crystal structures, with 1C3TA by MAFFT (71) shows conservation of E_51_ in 1C3TA with the catalytic E of the MMPs. The sequences have been represented as the MMP family_PDB ID. (C) Structural superimposition of the 3D-coordinates of the peptidase domain with the crystal structure of 1C3TA (I). Superimposition of 1AYK, of MMP1, peptidase domain (green) with 1C3TA (cyan). Panel II. Superimposition of 1A85, of MMP8, peptidase domain (green) with 1C3TA (cyan). Panel III. Superimposition of 1EUB, of MMP13, peptidase domain (green) on 1C3TA (cyan).

Sequence alignment between human ubiquitin and the placental ubiquitin (PDB code: 1C3T, chain: A) by clustalW indicated that apart from 8 substitutions on 1C3TA (residue numbers –3, 13, 15, 17, 23, 26, 61 and 67), the rest of the sequence is identical to human ubiquitin (Overall sequence identity = 89.5%; sequence similarity = 100%) (Figure 4A) [43]. The substitutions are also highly similar where I of human ubiquitin is replaced by L_3, 13, 61_ or V_23_; L_15,67_ is replaced by V_15_ or I_67_; and V_17,26_ are replaced by L_17,26_. Thus, I, L and V were primarily involved in the replacement with a reduction in hydrophobic character by 2.1 units in placental ubiquitin (total hydrophobicity of human ubiquitin is 34.0 while 1C3TA is 31.9 units) [44]. Thus, the reasons for thermal stability of placental compared to that of human ubiquitin could not be predicted from the alignment.

The MMPs in general are characterized by the presence of a peptidoglycan (PG) binding domain, a peptidase domain and a C-terminal hemopexin domain. MMP 2 and 9 contain an additional fibronectin domain and the PG binding domain in MMP 9 is replaced with a PT-repeat [45]. The complete primary sequences of each of the MMPs 1, 2, 3, 8, 9 and 13 when aligned with the sequence of 1C3TA, showed very poor sequence identities. The active and metal binding sites of MMPs 1, 2, 3, 8, 9 and 13 cluster together to form a conserved segment (H-E-F-G-H-[S/A]-[L/M]-G-L-X-H) within the catalytic domain [46]. However, when only the catalytic domains of the MMPs were aligned with 1C3TA, comparatively higher sequence similarity was observed indicating a certain degree of conservation. It is also to be noted that an active site E_51_ was found to be conserved in 1C3TA whereas a metal binding H_54_ is replaced by a similar amino acid, R (Fig 4B). The three dimensional (3D) co-ordinates of the MMP active and metal binding sites (alignment numbers: 297H, 298E, 301H and 307H; residue numbers: 218H, 219E, 222H and 228H with respect to 1AYK) were extracted from the corresponding PDB files of peptidases to perform a 3D motif scan against the 3D structure of ubiquitin (1C3TA) using spatial motif scanning softwares SPASM and RASMOT-3D PRO [47]-[49]. However, no significant structural similarities were established between MMPs and 1C3T via superimposition and 3D motif scan (Figure 4C, Table S1). These results suggest that the primary sequence of the catalytic domains of the respective MMPs and 1C3TA are relatively more conserved even though they posses overall low structural similarity. It appears that placental ubiquitin and collagenases progressed through the evolutionary process independent of one another that led to the formation of distinct catalytic domains altogether. Owing to a similar functional relationship, an overall convergent evolution is nonetheless indicative.

The finding that the ubiquitin-like component shows intrinsic collagenase activity *in vitro* might have implications in wound healing. However, the exact mechanism it employs in the process of healing and the kinetics and the regulation of its activity is yet to be determined. It had previously been reported that whether bound to or free, the intrinsic proteolytic activity of ubiquitin plays roles in many cellular events and owing to its ubiquitous nature; it might play diverse roles in different physiological conditions [38]. This finding, therefore, adds to one of the many non-traditional functions of ubiquitin.

## Materials and Methods

### Placental Extract

An extract of human placenta prepared from fresh term placentae using single hot and cold water was used. The drug-house M/s Albert David Ltd, Calcutta, supplied the extract as sold under the trade name ‘Placentrex’ [17]. The product was sterilized under saturated steam pressure of 15 psi at 120°C for 40 min and was routinely tested for HIV antibody and Hepatitis B surface antigen. It contains 0.2% benzoyl alcohol as preservative that does not interfere with the experiments presented here. Collection and handling of the placenta and manufacturing of the drug were done under the license of drug controlling authority. Pool of placental peptides was isolated as described earlier [19].

### Materials

The following fine chemicals and materials were obtained: tris-base (analytical grade) and sodium dodecyl sulfate from SRL, Bombay, India; acetonitrile (HPLC grade) from Spectrochem Pvt. Ltd., Mumbai, India; calcium chloride (fused) and glycerol from Qualigens, Mumbai, India; Triton X-100 from s d fine chem, Mumbai, India; gelatin (from bovine skin), acrylamide, BSA, collagen (acid soluble type III, calf skin), yeast alcohol dehydrogenase, myoglobin, carbonic anhydrase, bacterial collagenase (Clostridium histolyticum, type II), azoalbumin, azocasein, ubiquitin (bovine erythrocytes), fibronectin type IIIc, trypsin, lysozyme (chicken egg white), Freund’s adjuvant (complete and incomplete), urea, Dithiothreitol (DTT), DEAE-cellulose, iodoacetamide (IAA), α-cyano-4-hydroxycinnamic acid (CHCA), bromophenol blue, PMSF, anti-mouse IgG (whole molecule), alkaline phosphatase, nitrocellulose membrane, 5-bromo-4-chloro-3-indolyl phosphate (BCIP), nitro blue tetrazolium (NBT), amino-phenyl mercuric acetate (APMA), 3-aminopropyl-triethoxysilane (APTES) and ammonium bicarbonate from Sigma, USA; trifluoroacetic acid (TFA) from Thermo Fisher, USA; Immobiline non-linear immobilized pH graient (IPG strip, pH 3–10, 17 cm), sephadex G-15 and 3-[(3-cholamidopropyl) dimethylammonio]-1-propanesulfonate (CHAPS) from GE life sciences from Uppsala, Sweden; silver nitrate and acetic acid from Merck, USA; ubiquitin antibody (polyclonal) from Imgenex, USA; centricon nylon membrane YM-10 (cut-off limit 10 kDa) from Amicon, USA; C_18_ zip tip from Millipore, USA; EnzChek® gelatinase/collagenase assay kit from Molecular Probes, Invitrogen; Ampholytes (pH 3–10) from Bio-Rad; trypsin gold and trypsin from Promega, Madison, USA; casein from Spectrum Chemical, NJ, USA; mica sheets (ASTM grade ruby mica, size 20×20 MM, 0.27 to 0.33 mm thickness) from Mica Fab, Chennai, India; and human MMP 9 (breast cancer cell line MDA-MB-231 SFCM) and human MMP 2 (breast cancer cell line MCF7 SFCM) from Chittaranjan National Cancer Institute, Kolkata, India (kindly gifted by Dr. Amitava Chatterjee, [50], [51]).

### Gelatin/Collagen Zymography

Gelatin was incorporated at 1.5 mg/ml after solubilizing in water at boiling temperature prior to polymerization with 12.5% polyacrylamide containing 0.1% SDS. Zymogram was developed after washing with 2.5% (v/v) Triton-X 100, followed by incubation at 37°C for 22 h in 50 mM Tris-HCl, pH 7.6 containing 5 mM CaCl_2_. Finally, staining with Coomassie blue and destaining yielded dark blue background against unstained regions of protease-digested gelatin [52]-[54]. Collagen zymography was essentially similar to that of gelatin zymography as mentioned above. Collagen was dissolved in 0.1 M acetic acid at 4°C to 15 mg/ml. It was then incorporated at 1.5 mg/ml prior to polymerization with 10% solution of polyacrylamide containing 0.1% SDS [55].

### SE-HPLC

Collagen treated with collagenase and placental extract at different time durations (0–168 h) was analyzed using Protein Pak 125 column (Waters, 13×78 mm, fractionation range 5–80 kDa). The column was equilibrated with 10 mM Na-phosphate, pH 7.5 containing 100 mM NaCl at 0.8 ml/min and elution was followed at 220 nm. The column was precalibrated with the following marker proteins, lysozyme (14 kDa), myoglobin (19 kDa), trypsin (22 kDa), carbonic anhydrase (31 kDa), ovalbumin (45 kDa), BSA (67 kDa) and yeast alcohol dehydrogenase (150 kDa). A linear dependency was observed between log M_w_ and retention time (R^2^ = 0.942).

### Dot Blot (Immuno-blot)

Placental peptide fraction (30 µg), collagenase (10 µg), ubiquitin (15 µg), and non-specific protein such as ovalbumin or lysozyme (10 µg) were spotted on nitrocellulose membrane strips, dried and incubated with PBS containing 0.1% Tween-20 and 5.0% skimmed milk at 4°C for 18 h. The strips were washed four times with PBS followed by incubation with placental peptide antibody (1∶1000 dilution) or ubiquitin antibody (1∶2000 dilution) for 2 h [56], [57]. The rest of the protocol was followed as described in [19].

### 2D-gel Electrophoresis

Placental extract (100x) was treated with 0.2 ml rehydration buffer (8 M urea, 2% CHAPS, 50 mM DTT, 0.2% ampholytes pH 3–10 and 0.002% bromophenol blue). Electrophoresis was carried out following the manufacturer’s protocol (Bio-Rad Laboratories, Inc, USA). Briefly, non linear pH 3–10 IPG strip was rehydrated with 0.25 ml rehydration buffer containing 0.05 ml sample overnight at 25°C and then subjected to isoelectric focusing. The strip was then equilibrated with reducing buffer (6 M urea, 2% SDS, 30% glycerol, 50 mM Tris-HCl and 25 mM DTT) followed by alkylation buffer (6 M urea, 2% SDS, 30% glycerol, 50 mM Tris-HCl and 135 mM IAA). The strip was then placed on 12.5% SDS polyacrylamide gel using 1% agarose in SDS-PAGE running buffer. After electrophoresis, the gel was washed thoroughly with water, fixed overnight with 40% methanol and 10% acetic acid; which was then stained with silver nitrate [58]. Gelatin zymography was performed under denaturing but non-reducing conditions.

### MALDI-TOF MS

Protein spots were excised and sliced into 1 mm cube, dehydrated in acetonitrile and dried in vacuum [59]. NH_4_HCO_3_ (100 mM containing 20 mM DTT, 0.05 ml) sufficient to cover the gel pieces was added and the protein was reduced for 1 h at 60°C. After cooling to around 25°C, the solution was replaced by an equal volume of 100 mM NH_4_HCO_3_ containing 55 mM IAA and incubated for 45 min at 25°C in the dark. The gel pieces were rinsed thrice with 100 mM NH_4_HCO_3_ and acetonitrile successively using vortex. The liquid phase was removed, the gel pieces were completely dried in vacuum and swollen in 0.025 ml digestion buffer (100 mM NH_4_HCO_3_, 5 mM CaCl_2_ and 1 µg trypsin gold) in ice bath for 1 h after which another 0.025 ml of digestion buffer was added and incubated overnight at 37°C. The supernatant containing peptides in ammonium bicarbonate was extracted, concentrated and was maintained in 50% acetonitrile/0.1% TFA for MS/MS analysis.

A 4800 MALDI TOF/TOF™ (Applied Biosystems) operated in reflectron mode was used. Peptide mixture was desalted using C_18_ zip tip and analyzed using a saturated solution of CHCA in 50% acetonitrile/0.1% TFA. The MS/MS peak of the most intense tryptic peptide mass ion peak were searched against Swissprot and NCBInr database using Mascot (Matrix Science, Ltd., London, United Kingdom; http://www.matrixscience.com) search program with fixed and variable modifications; Carbamidomethyl (C) and Oxidation (M) respectively.

### Gelatinase/Collagenase Assay

Enzyme activity was investigated with EnzChek® Gelatinase/Collagenase assay kit (Molecular Probes Inc.). Samples were incubated with DQ-gelatin in dark at 25°C. Active enzymes cleave the fluorescent-labeled gelatin and change in fluorescence was measured (ex/em 495/515 nm). The reaction was followed for 30 min after addition of DQ gelatin to the peptide fraction, placental extract or ubiquitin as applicable, pre-incubated at 37°C between 14–92 h. Slit width was adjusted to 5/5 nm (ex/em). For the inhibition assay, prior to addition of DQ-gelatin, the preincubated enzyme was incubated for 10 min with ubiquitin antibody at 25°C, following which the assay was initiated by the addition of DQ-gelatin as mentioned above.

Standard assay using freshly prepared collagenase (0.012–0.096 U/ml) in 20 mM Tris-HCl (pH 7.6) containing 50 mM CaCl_2_ and DQ-gelatin (12.5 µg) indicated increase in activity with increasing concentrations of the enzyme, till at saturation no further increase in fluorescence was observed. A plot of collagenase concentration versus slope of the reaction (rate) indicated a straight line with R^2^ = 0.944, where R^2^ is the regression coefficient.

### AFM

Collagen was initially dissolved at 2 mg/ml in 0.1 M acetic acid, pH ∼ 3.7 at 4°C. Freshly cleaved mica sheets were treated with APTES for 2 hr by evaporation method [60], [61]. 0.25 ml collagen (0.1 mg/ml in 10 mM Na-phosphate, pH 7.5) was incubated in the APTES treated mica sheet for 2 h. Collagen (0.1 mg/ml) treated with collagenase (1 µg/ml), peptide fraction (50 µg/ml) or ubiquitin (50 µg/ml) at 37°C for 48 h was further incubated for 2 hr in freshly cleaved mica sheet without APTES treatment, owing to change in overall surface charge.

The adsorbed collagen assemblies (treated or untreated) were imaged in liquid by Acoustic Alternative Current or AAC (Tapping) Mode using a Picoplus AFM 5500 (Agilent technologies, USA). SuperSharpSilicon Non-Contact Low frequency or SSS-NCl (Nanosensors, Switzerland) long cantilevers (thickness 7±1 µm, length 225±10 µm, tip radius of 2 nm; with a force constant between 21–98 N/m and resonance frequency between 146–236 kHz) were used. The scan frequency was set at 1.5 Hz. A 9 or a 100 µm scanner was used as applicable. All images were analyzed using Molecular Imaging Corporation PicoScan 5.3.3 (Molecular Imaging Corporation, USA).

### Alignment and Structural Superimposition

Multiple sequence alignment between MMPs and ubiquitin was performed by MAFFT whereas structural superimposition was done using MUSTANG [62], [63]. SPASM and RASMOT-3D PRO were used to scan 3D motif [48], [49], [64].

### Other Methods

Fluorescence measurements were done using a Hitachi F 4500 or F 7000 instrument having ex/em slit widths of 5 nm each. Optical absorbances were recorded with a Specord 200 spectrophotometer (Analytic Jena, Germany). Placental peptide antibody was isolated as described in [19].

## Supporting Information

Figure S1 **Gelatinase/collagenase assay.** (A) Reaction kinetics of collagenase activity of peptide fraction in absence (a) and presence (b) of ubiquitin antibody. (n = 5). (B) Activation profile of gelatinase/collagenase activity of peptide fraction up to 72 h (n = 3).
(TIF)

Figure S2 **Change of solubility of collagen in buffer (control) or in presence of collagenase, ubiquitin (Sigma) and placental extract.** The hours of incubation is indicated (n = 3).
(TIF)

Table S1 **Root mean square deviation (RMSD) values obtained after superimposition of 1CT3A with the peptidase domains of the matrix metalloproteases belonging to the collagenase family.**
(DOC)
