# Supplementary figures and images for: Ubiquitin-Like Protein from Human Placental Extract Exhibits Collagenase Activity
Source: PLoS One. 2013 Mar 26;8(3):e59585. doi: 10.1371/journal.pone.0059585 (PMC3608664; doi:10.1371/journal.pone.0059585)

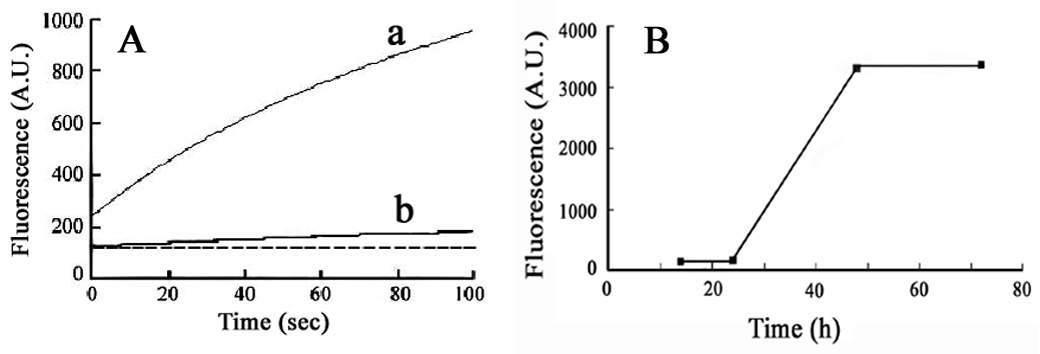

Supplement: Figure S1 — Gelatinase/collagenase assay. (A) Reaction kinetics of collagenase activity of peptide fraction in absence (a) and presence (b) of ubiquitin antibody. (n = 5). (B) Activation profile of gelatinase/collagenase activity of peptide fraction up to 72 h (n = 3). (TIF) [file pone.0059585.s001.tif]

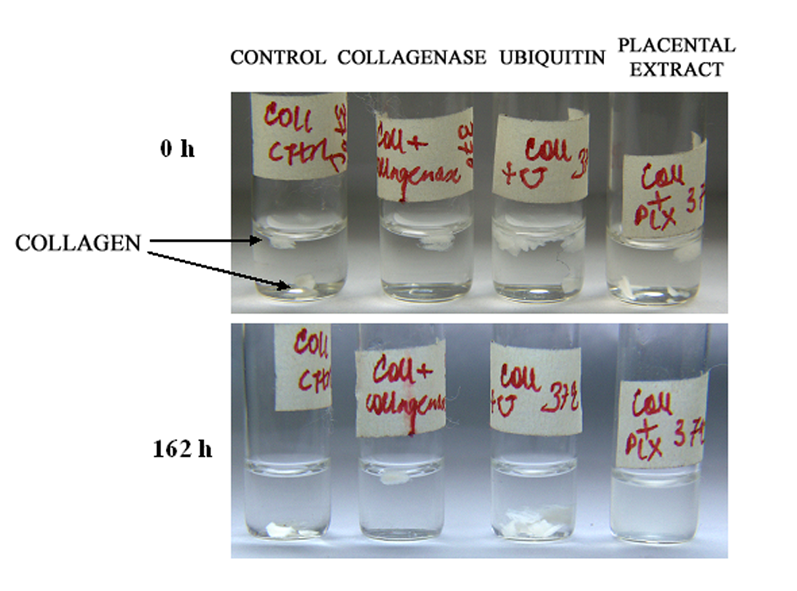

Supplement: Figure S2 — Change of solubility of collagen in buffer (control) or in presence of collagenase, ubiquitin (Sigma) and placental extract. The hours of incubation is indicated (n = 3). (TIF) [file pone.0059585.s002.tif]
