# Supplementary material for: Ubiquitin-Like Protein from Human Placental Extract Exhibits Collagenase Activity
Source: PLoS One. 2013 Mar 26;8(3):e59585. doi: 10.1371/journal.pone.0059585 (PMC3608664; doi:10.1371/journal.pone.0059585)
Supplement: Table S1 — Root mean square deviation (RMSD) values obtained after superimposition of 1CT3A with the peptidase domains of the matrix metalloproteases belonging to the collagenase family. (DOC) [file pone.0059585.s003.doc]

| **PDB Structures of Metallopeptidases Peptidase Domain** | **RMSD with 1C3T (A°)** | **Structural Identity**  **(%)** |
| --- | --- | --- |
| 1AYK (MMP 1) | 12.6 | 0.8 |
| 1CK7 (MMP 2) | 14.8 | 1.1 |
| 1A85 (MMP 8) | 12.7 | 2.9 |
| 1L6J (MMP 9) | 15.4 | 1.7 |
| 1EUB (MMP 13) | 12.2 | 2.4 |

**Table S1. Root mean square deviation (RMSD) values obtained after superimposition of 1CT3A with the peptidase domains of the matrix metalloproteases belonging to the collagenase family.**
